# Down-regulation of SNHG16 alleviates the acute lung injury in sepsis rats through miR-128-3p/HMGB3 axis

**DOI:** 10.1186/s12890-021-01552-0

**Published:** 2021-06-06

**Authors:** Junli Sun, Keke Xin, Chenghui Leng, Jianlin Ge

**Affiliations:** General ICU, Luoyang Central Hospital Affiliated To Zhengzhou University, 288 Zhongzhou Middle Road, Luoyang, 471009 Henan China

**Keywords:** Sepsis, Acute lung injury, SNHG16, Inflammatory response, Apoptosis

## Abstract

**Background:**

Long noncoding RNAs contribute to various inflammatory diseases, including sepsis. We explore the role of small nucleolar RNA host gene 16 (SNHG16) in sepsis-mediated acute lung injury (ALI) and inflammation.

**Methods:**

A sepsis-induced ALI rat model was constructed by the cecal ligation and perforation method. The profiles of SNHG16, miR-128-3p, and high-mobility group box 3 (HMGB3) were monitored by quantitative reverse transcription PCR and Western blot. The pathologic changes of lung tissues were evaluated by Hematoxylin–Eosin staining, immunohistochemistry, and dry and wet method. Meanwhile, the pro-inflammatory factors and proteins were determined by ELISA and Western blot. In contrast, a sepsis model in BEAS-2B was induced with lipopolysaccharide (LPS) to verify the effects of SNHG16/miR-128-3p/HMGB3 on lung epithelial cell viability and apoptosis.

**Results:**

As a result, SNHG16 and HMGB3 were up-regulated, while miR-128-3p was down-regulated in sepsis-induced ALI both in vivo and in vitro*.* Inhibiting SNHG16 reduced the apoptosis and inflammation in the sepsis-induced ALI model. Overexpressing SNHG16 promoted LPS-mediated lung epithelial apoptosis and inhibited cell viability and inflammation, while miR-128-3p had the opposite effects. Mechanistically, SNHG16 targeted miR-128-3p and attenuated its expression, while miR-128-3p targeted the 3′ untranslated region of HMGB3.

**Conclusions:**

Overall, down-regulating SNHG16 alleviated the sepsis-mediated ALI by regulating miR-128-3p/HMGB3.

**Supplementary Information:**

The online version contains supplementary material available at 10.1186/s12890-021-01552-0.

## Background

Sepsis is an overreaction of the host to severe infection, which mainly causes dysfunction of vital organs and is one of the common causes of death in hospitals, especially in the intensive care unit (ICU) [1]. The lung is the first organ that responds to sepsis stimulation, and about 50% of acute lung injury (ALI) is related to sepsis [2]. ALI is a pulmonary manifestation of systemic inflammation, which is mainly characterized by severe hypoxemia and immune cells infiltration in lung, which often result in death because of acute respiratory distress [3]. The current treatment of ALI is based on antibiotics and supportive therapy, while the curative effect is very limited, and the mortality rate after treatment remains high. Therefore, research on effective biomarkers is essential for early diagnosis and timely treatment of ALI.

Long noncoding RNAs (lncRNAs) are endogenous RNAs with a length greater than 200 bp. Although lack of protein-coding ability, lncRNAs are of great significance for treating malignant tumors [4]. In addition, multiple lncRNAs have shown excellent resistance to sepsis-induced organ damage. For example, Wang et al. showed that lncRNA NEAT1 eases the lipopolysaccharide (LPS)-induced myocardial damage in mice by inhibiting TLR2/NF-κB [5]. Besides, Jiang et al. found that lncRNA HOTAIR alleviates ALI in septic rats by inhibiting the miR-34a/Bcl-2 axis [6]. Small nucleolar RNA host gene 16 (SNHG16) is a type of lncRNA located on chromosome 17q25.1. Studies have shown that SNHG16 serves as an oncogene in non-small cell lung cancer (NSCLC) [7]. A recent study indicated that SNHG16 promoted TLR4 expression by targeting miR-15a/16, thus repressing the anti-inflammatory effects mediated by miR-15a/16 [8]. More importantly, knocking down SNHG16 inhibits the CCL5 expression by up-regulating miR-146a-5p, thereby mitigating LPS-induced lung cell damage, which is helpful for the diagnosis and treatment of acute pneumonia [9]. Additionally, SNHG16 aggravates LPS-induced acute pneumonia in A549 cells through modulating miR-370-3p/IGF2 axis [10]. Therefore, those studies suggest that SNHG16 makes a great contribution in LPS-mediated inflammation, especially in lung injury.

MicroRNAs (miRNAs) are small double-stranded RNAs of 19–25 nucleotides in length. They regulate post-transcriptional gene expression through translation inhibition or degradation enhancement of messenger RNAs (mRNAs), which affects biological processes such as cell proliferation, differentiation and apoptosis [11]. MiRNAs are not only abnormally expressed in lung cancer, but also contribute to regulating other lung diseases. For instance, some scholars have found that miR-150 reduces LPS-induced ALI by targeting AKT3 or inhibiting the JNK/NF-κB pathway [12]. What’s more, Ju et al. stated that miR-27a reduces inflammatory stimulation by inactivating TLR4/MyD88/NF-κB, thereby relieving LPS-induced ALI in mice [13]. MiR-128-3p is a newly discovered miRNA in recent years. Studies by Pan et al. illustrated that miR-128-3p is knocked down in lung cancer tissues and cell lines, which is helpful for the diagnosis of early lung cancer [14]. Moreover, miR-128-3p facilitates the protection of dexmedetomidine against ALI in sepsis mice by dampening MAPK14 [15]. Hence, miR-128-3p has the potential effect in preventing lung injury.

High Mobility Group Box (HMGB), including HMGB1, HMGB2, HMGB3 and HMGB4, is an extracellular endogenous molecule released after tissue injury [16]. HMGB3 is mainly distributed in the nucleus, cytoplasm and chromosomes, and has 85–89% homology with HMGB1 and HMGB2 [17]. Among the four members of HMGB, HMGB1 is considered as a potential therapeutic target for sepsis, and glycyrrhizin protects rats from sepsis-induced damage by preventing the HMGB1-mediated inflammation [18]. Ueno et al. found that HMGB2 was upregulated in LPS-treated mice, and it was also positively expressed in the epithelial lining fluid and serum of ARDS/ALI patients [19]. HMGB4 is expressed by neuronal cells and affects the expression of genes involved in neural differentiation [20]. As for HMGB3, it plays a carcinogenic role in glioblastoma [21] and NSCLC [22], while its effect on sepsis-related diseases is scarcely reported.

Overall, the present studies indicate that SNHG16 is involved in lung injury by targeting miRNAs and regulating downstream molecules, and miR-128-3p has potential effects in alleviating ALI in septic mice. Interestingly, our study found that SNHG16 and HMGB3 were up-regulated in sepsis model with ALI, while miR-128-3p was downregulated. Therefore, we speculate that there’s a regulatory axis of SNHG16/miR-128-3p/HMGB3 in sepsis-mediated ALI.

## Methods

### The rat model

Total Eighty male Sprague–Dawley rats (180–220 g, 6–8 weeks old) were purchased from the Animal Experiment Center of Huazhong University of Science and Technology (Wuhan, China). The rats were randomly divided into four groups (n = 20 in each group). The rats were maintained at 22–24 °C with 50–60% humidity and 12 h of light/dark cycle for one week, during which adequate food and water were given, and then cecal ligation and puncture (CLP) was performed to induce sepsis in rats. Preoperative fasting was applied to the rats. Afterward, pentobarbital sodium (50 mg/kg) was injected intraperitoneally to anesthetize the rats. After disinfection, the abdominal skin was incised for about 2 cm, and the cecum was gently pulled out with sterile forceps. The roots of the cecum were ligated with a 3–0 silk thread, and the head and tail of the cecum were perforated with a 20-G sterile needle. The two perforations were about 1 cm apart. Subsequently, a small amount of stool was gently squeezed out, and the cecum was repositioned in the abdominal cavity, and then the muscle, peritoneum and skin were sutured. Immediately after the operation, the rats were resuscitated by the subcutaneous injection of saline. E. coli counts in the bronchoalveolar lavage fluid (BALF) and peritoneal fluid were determined. The sham group did not undergo CLP, and the remaining steps were the same as the experiment group. In the CLP + sh-NC and CLP + sh-SNHG16 groups, rats were injected with 100 µl sh-NC or sh-SNHG16 (10 µM) (RiboBio Co., Ltd, Guangzhou, China) through the tail vein 48 h before CLP was performed. Finally, the rats were euthanized and the lung tissues were obtained and preserved in liquid nitrogen at − 196 °C for follow-up experiments. This experiment was carried out by observing the "Guidelines for the Care and Use of Laboratory Animals" (NIH Publication No. 85-23, 2011) published by the National Institutes of Health. All of the animal procedures had been approved by the medical ethics committee of Luoyang Central Hospital [approve number: SCXK(E)2016-0009] and also carried out in compliance with the ARRIVE guidelines (http://www.nc3rs.org.uk/page.asp?id=1357).

### Cell culture and transfection

Human normal lung epithelial cell line BEAS-2B was purchased from American Type Culture Collection (ATCC, Rockville, MD, USA) and cultured in the DMEM medium (Thermo Fisher HyClone, Utah, USA) containing 5% FBS (Thermo Fisher Scientific, MA, USA) at 37 °C with 5% CO_2_. Then, the cells were incubated with LPS (Sigma, Shanghai, China) at different doses (100 ng/mL) for 24 h.

The well-grown BEAS-2B cells were taken and inoculated into 6-well plates at 5 × 10^5^/well. When the cells reach a fusion rate of 80–90%, they were transfected with 100 μL SNHG16 overexpression (SNHG16)/knockdown (sh-SNHG16) plasmids and miR-128-3p mimics (miR-128-3p) (300 nM) following the Lipofectamine 2000 reagent instructions (Invitrogen; Thermo Fisher Scientific, Inc., Waltham, MA, USA). The untreated cells served as control.

### Quantitative reverse transcription PCR (qRT-PCR)

TRIzol (Invitrogen, Carlsbad, CA, USA) method was adopted to collect the total RNA from BEAS-2B cells and rat lung tissues, and the RNA was reversely transcribed into cDNA by referring to the instructions of the TOYOBO Reverse Transcription Kit (TOYOBO, Osaka, Japan). The reaction conditions for RT includes: total RNA (2 μg in DEPC H_2_O (11 μL) and 14 μL oligo (DT) (10 μM) were mixed, 70 ℃ for 10 min, 1 ℃ for 1 min; then the first strand cDNA 2 μL, forward primer 2 μL, reverse primer 2 μL, dNTP (2 mm) 4 UL, 10 × PCR buffer 5 μL, Taq enzyme 1 μL were mixed, 42 ℃ for 50 min, 70 ℃ for 15 min to terminate the reaction, incubate at 37 ℃ for 20 min to degrade the residual RNA. qRT-PCR was implemented with the SYBR® Premix Ex Taq™ II Kit (Takara, Otsu, Japan), with GAPDH as the endogenous control for SNGG16 and HMGB3, while U6 was for the internal control of miR-128-3p. The fluorescent quantitative PCR system was 25 μL, containing 2 μL cDNA template, 250 nmol/L upstream and downstream primers and 12.5 μL 2 × SYBR® Premix. The reaction condition includes: 95 °C for 10 min, 40 cycles of 95 °C for 15 s and 60 °C for 1 min, 40 cycles. The primer sequences were shown in Table 1. The relative expression was calculated with the 2^−ΔΔCT^ method.

**Table 1:** Primer sequence of each molecule

Name	Primer sequences
SNHG16	Forward: 5′-GTTCCTCTAAAAAAAAGCGCCATGCGTTCT-3′
	Reverse: 5′-CATTTCAGTTTACAATCCTTGCAGTCCC-3′
miR-128-3p	Forward: 5′-GACTGCCGAGAGCGAGCG-3′
	Reverse: 5′-GACGCCGAGGCACTCTCTCCT-3′
HMGB3	Forward: 5′-ATTCGGAATTCCGTATCTGGCCTTTTGAC-3′
	Reverse: 5′-CGGTTACTCGGCTTACGCTTGGACTG-3′
GAPDH	Forward: 5′-TGGTTGAGCACAGGGTACTT-3′
	Reverse: 5′-CCAAGGAGTAAGACCCCTGG-3′
U6	Forward: 5′-GTGCAGGGTCCGAGGT-3′
	Reverse: 5′-CTCGCTTCGGCAGCACA-3′

### Hematoxylin–eosin (HE) staining

The lung, liver, kidney and spleen tissues of the rat described in method 2.1 was fixed with 4% paraformaldehyde for 24 h, paraffin-embedded and sectioned (4 μm). Then, the sections were dehydrated with gradient alcohol, cleared with xylene, and mounted with resin. Then the sections were respectively stained with hematoxylin and eosin solution (Beyotime, Shanghai, China). Finally, the pathological changes of lung tissue were monitored with the microscope (Olympus BX 53 microscope, Tokyo, Japan).

### Immunohistochemistry (IHC)

The lung tissues of sepsis rats were conventionally paraffin-embedded, sectioned (5 μm), dewaxed with xylene, hydrated with gradient alcohol and blocked with 3% H_2_O_2_ for 10 min to inactivate the endogenous peroxidase. After the sections were blocked with 5% bovine serum albumin for 20 min, they were incubated with the primary antibody Anti-Caspase-3 antibody (1:100, ab32351, Abcam, MA, USA) or anti-MPO antibody (1:100, ab208670, Abcam, MA, USA) at 4℃ overnight. After the sections were washed by PBS for 3 times (10 min each time), the secondary antibody Goat Anti-Rabbit IgG (1:500, ab150077, Abcam, USA) was added dropwise and incubated with the sections at room temperature (RT) for 20 min. Then, the sections were dehydrated, cleared, and fixed on a glass slide with neutral resin. At last, the sections were observed with a Nikon microscope (Nikon, Japan).

### Wet-to-dry (W/D) method

The middle lobe of the right lung of experimental rats was taken, and the wet weight (W) was recorded after the surface water was sucked dry. Then the right middle lobe of the lung was heated in a constant temperature oven at 80 °C for 48 h to constant weight, weighed again and recorded the as the dry weight (D). The pulmonary edema index = W/D.

### Arterial blood gas analysis

0.5 mL arterial blood of the rats was drawn from the abdominal aorta of a sepsis rat with a syringe supplemented with heparin and immediately injected into a blood gas analyzer (Beckman Coulter, Inc., USA) immediately to determine the arterial partial pressure of oxygen (PaO_2_), carbon dioxide (PaCO_2_) and pH value.

### Enzyme-linked immunosorbent assay (ELISA)

The rat lung tissues were collected, then 200 μL PBS was used for tissue homogenate (per 100 mg tissue). The culture medium of BEAS-2B cells after treatments was also collected by centrifugation for removing cells or debris. The levels of IL-1β (RLB00 or DLB50), IL-6 (R6000B or HS600C), and TNF-α (RTA00 or HSTA00E) in CLP rat lung homogenate and culture medium were determined with ELISA kits following the supplier's guidelines (R&D Systems, Minneapolis, MN, USA). Then, the OD was observed with a Power Wave microplate reader (Bio-TEK, USA) at 450 nm.

### Immunofluorescence

BEAS-2B cells were transfected with SNHG16 and/or miR-128-3p mimics. The stably-transfected cells were seeded in 24-well plates (1 × 105 cells per well). 24 h later, the cells were fixed with 4% paraformaldehyde at 25 °C for 30 min, and then permeabilized with 0.3% Triton X-100 for 20 min. Followed by that, the cells were blocked with 5% BSA (at room temperature for 1 h), and incubated with primary anti-HMGB3 antibody (ab75782, Abcam, USA) at 4 °C for 12 h. The next day, the cell were washed by PBS and incubated with Goat polyclonal Secondary Antibody to Rabbit IgG—H&L (Alexa Fluor® 488) at 4 °C for 1 h. After being washed with PBS, DAPI solution (10 μg/mL) was used for staining the nuclear for 10 min in the dark. Finally, a confocal laser scanning microscope (BIO-RAD, Hemel Hempstead, UK) was used for observing the cell images.

### Western Blot (WB)

The total protein of lung tissues and BEAS-2B cells were extracted and then quantified with bicinchoninic acid assay (Pierce, Rockford, USA). Then, it was resolved on 10% polyacrylamide gel electrophoresis and transferred onto polyvinylidene fluoride (PVDF) membranes. Afterward, the PVDF membranes were blocked with 5% skim milk at RT for 1 h and incubated with the primary Anti-NF-κB p65 (phospho S276) antibody (1:1000, ab222494, Abcam, USA), Anti-NF-κB p65 antibody (1:1000, ab16502, Abcam, USA), Anti-MAPK p38 (phospho T180) antibody(1:1000, ab178867, Abcam, USA), Anti-MAPK p38 antibody(1:1000, ab31828, Abcam, USA), Anti-iNOS antibody(1:1000, ab178945, Abcam, USA), Anti-COX2 antibody (1:1000, ab179800, Abcam, USA), Anti-HMG4 antibody (1:1000, ab75782, Abcam, USA), Anti-β-actin antibody (1:1000, ab8224, Abcam, USA) and Anti-GAPDH antibody (1:1000, ab181602, Abcam, USA) overnight at 4 °C. Subsequently, the membranes were washed twice with TBST, and then incubated with the fluorescein-labeled secondary Goat Anti-Rabbit IgG (1:3000, ab150077, Abcam, USA) at RT for 1 h. After that, the membranes were rinsed 3 times. The protein bands were examined by the ECL WB kit (Amersham Biosciences, UK). The primary images of the blots were shown in the Additional file 1: Fig. S1, Additional file 2: Fig. S2 and Additional file 3: Fig. S3.

### Dual-luciferase reporter assay

The BEAS-2B cells were inoculated in 48-well plates at 5 × 10^5^/well and cultured to 70% confluence. Then, the cells were transfected with SNHG16-WT, SNHG16-MUT, HMGB3-WT, HMGB3-MUT (0.5 μg) and miR-128-3p mimics and their negative controls (300 pmol) with the liposome 2000 reagent (Invitrogen; Thermo Fisher Scientific, Inc., Waltham, MA, USA). After 48 h, the luciferase activity was monitored as per the supplier's instructions (Promega, Madison, WI, USA).

### RNA immunoprecipitation (RIP) assay

RIP assay was carried out with the Magna RipRNA binding protein IP kit (Millipore, Bedford, MA, USA) following the kit’s protocol. The complete RIP lysis buffer was employed to lyse BEAS-2B cells, and then 100 μL of whole-cell extract was incubated with the RIPA buffer containing magnetic beads coupled with the human anti-Argonaute2 (Ago2) antibody (Abcam, Shanghai, China) at 4 °C for 6 to 8 h. Immunoglobulin G (IgG) antibody served as a negative control. Finally, RNA was extracted with the TRIzol reagent (Invitrogen, Carlsbad, CA, USA), reversely transcribed into cDNA, and analyzed by qRT-PCR.

### 3-(4, 5-dimethylthiazol-2-yl)-2, 5-diphenyltetrazolium bromide (MTT) assay

Stably transfected BEAS-2B cells were inoculated in 96-well plates at 2 × 10^4^/well and incubated at 37 °C with 5% CO_2_ for 24 h. Different factors were administered to treat the cells. The blank control group was supplemented with an equal volume of PBS, and 5 repetitive wells were set in each group. After culturing for 24 h, the cells were incubated with 20 μL MTT solution (5 mg/mL, Beyotime, Shanghai, China) at 37℃ for 4 h. After that, the supernatant was aspirated and DMSO was added to lyse the cells. After the crystals were dissolved, the OD value of each well was observed with a microplate reader (Bio-Rad, Hercules, CA, USA) at 570 nm.

### Flow cytometry

AnnexinV/7-AADA apoptosis Detection Kit (Southern Biotechnology, Birmingham, Al, USA) was adopted to determine cell apoptosis following the kit instructions. The treated BEAS-2B cells were trypsinized and centrifuged, and then washed twice with cold PBS. Afterward, the cells were double-stained with AnnexinV and propidium iodide (PI) and incubated in the dark at RT for 15 min. Finally, the FACScan flow cytometer (FCM; Bechman Coulter, CA) was used to analyze apoptosis.

### Statistical analysis

GraphPad Prism 8 (GraphPad Software, Inc., city, state) was employed to analyze statistical differences. The measurement data were presented as mean ± standard deviation (${\overline{\text{x}}} \pm {\text{s}}$). *t* test was used for pairwise comparison, and the comparison of multiple samples was made by one-way ANOVA. *P* < 0.05 indicated statistical significance.

## Results

### Expression of SNHG16/miR-128-3p/HMGB3 in the sepsis-mediated ALI rat model

**Fig. 1:**
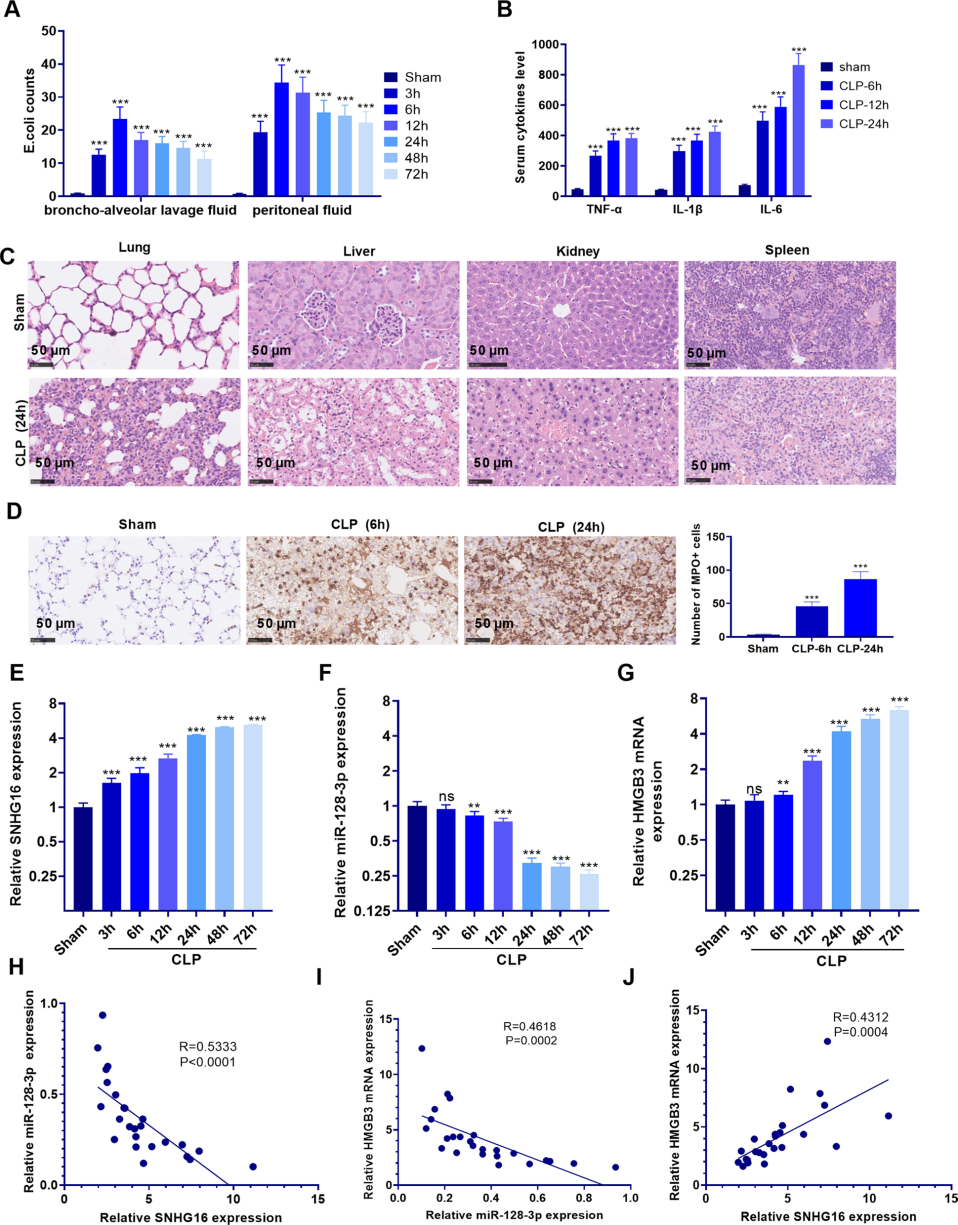
The expression of SNHG16/miR-128-3p/HMGB3 in a rat model of sepsis-induced ALI. Rats were treated with CLP to establish a sepsis model. **a** *E. coli* counts in the bronchoalveolar lavage fluid (BALF) and peritoneal fluid were determined by in-vitro bacterial culture. **b** ELISA was conducted to determine the inflammatory cytokines including IL-1β, IL-6 and TNF-α in the lung tissue homogenate. **c** HE staining was used for detecting the histopathological changes of lung, kidney, liver and spleen. **d** IHC was used for determining Neutrophil infiltration (labeled by MPO) in the lung. N = 5. **e**–**g** qRT-PCR was employed to examine the expression of SNHG16, miR-128-3p, HMGB3 in the lung tissue of septic rats at different time points (sham, 3, 6, 12, 24, 48, 72 h). NS *P* > 0.05; **P < 0.01; ***P < 0.001. **h**–**j** Linear regression analysis was used to analyze the relationships among SNHG16, miR-128-3p and HMGB3 in the lung tissue of septic rats at 24 h after CLP. N = 25. All experiments were repeated for three times

We constructed a rat ALI model through CLP to study the expression of each molecule. First, we detected *E. coli* counts in the BALF and peritoneal fluid. The result showed that significantly increased *E. coli* was found the rats in the CLP group (not in the sham group) (Fig. 1a). And the ELISA data confirmed that those inflammatory cytokines (including IL-1β, IL-6 and TNFα were all enhanced in the lung of CLP group (compared with those in the CLP group) (Fig. 1b). And HE staining showed that the rats in the CLP group were acquired with significant lung, liver, kidney injuries, as there were obvious blood barrier damage and tissue edema (Fig. 1c). What’s more, the results of ICH confirmed that the prominent MPO-positive neutrophils were infiltrated in the lungs of the CLP group (Fig. 1d). Those results indicated a sepsis-ALI rat model was successfully constructed. Next, qRT-PCR was conducted to analyze the SNHG16/miR-128-3p/HMGB3 expression in sepsis-mediated ALI rats. It was found that the expression of SNHG16 and HMGB3 in the lung tissue of the CLP group increased with time (compared with the Sham group), while miR-128-3p was decreased in the CLP group (*P* < 0.05, Fig. 1e–g). Furthermore, we conduct linear regression analysis to explore the correlations among SNHG16, miR-128-3p and HMGB3. It suggested that the levels of SNHG16 and HMGB3 were positively correlated in septic rats, while miR-128-3p was inversely correlated with SNHG16 and HMGB3 (Fig. 1h–j). Therefore, the above data suggested that all of SNHG16, miR-128-3p and HMGB3 play a role in sepsis-mediated ALI.

### The SNHG16 knockdown reduced sepsis-induced ALI

**Fig. 2:**
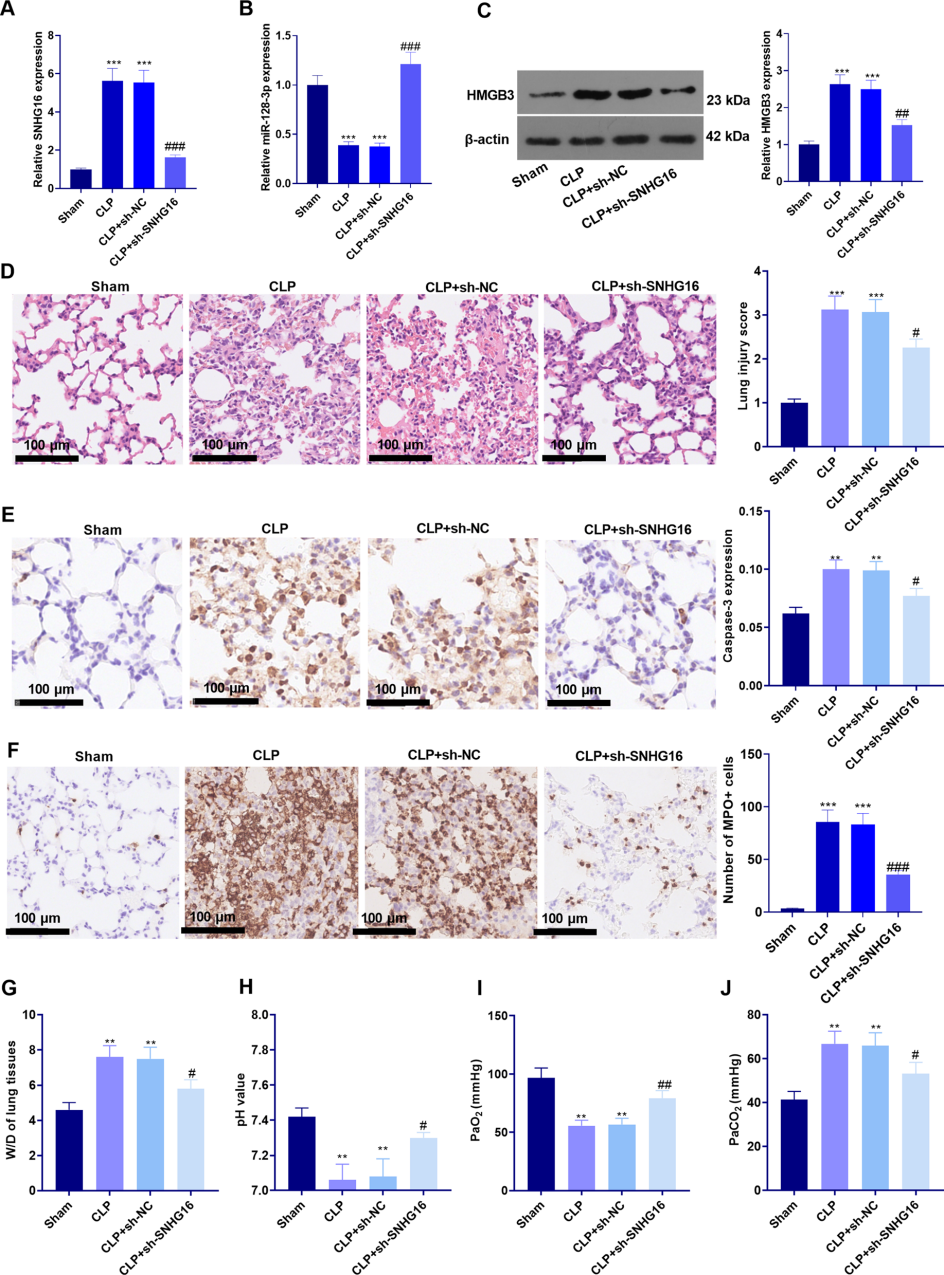
SNHG16 knockdown attenuated sepsis-induced ALI. sh-SNHG16 was used for establishing a SNHG16-knockdodwn rats, which were then subjected to CLP to induce a sepsis model. **a**, **b** qRT-PCR was employed to examine the expression of SNHG16 and miR-128-3p in the lung tissue of rats. **c** Western blot was employed to examine the expression of HMGB3 in the lung tissue of rats. **d** HE staining was performed to observe the lung pathological changes of rat lung tissues. **e**, **f** IHC was implemented to monitor the Caspase3 (**e**) and MPO (**f**) expression in the rat lung tissue; G: The pulmonary edema was determined with the W/D method; **h**, **j** The arterial blood of rats was adopted for blood gas analysis to verify the changes in lung function. **P < 0.01; ***P < 0.001 (vs. Sham group); ^#^*P* < 0.05; ^##^*P* < 0.01; ^###^*P* < 0.001 (vs. Sham group). N = 5. All experiments were repeated for three times

In order to clarify the specific effect of SNHG16 in sepsis-mediated ALI, we constructed a rat sepsis model through CLP and then injected sh-NC and sh-SNHG16 into the rats through their tail veins. qRT-PCR and western blot results showed that compared with the CLP + sh-NC group, SNHG16 and HMGB3 were both downregulated in the CLP + sh-SNHG16 group, while miR-128-3p was enhanced in the CLP + sh-SNHG16 group (Fig. 2a–c). HE staining showed that the CLP rats were with severely damaged alveoli and congested capillaries after CLP (Fig. 2d). What’s more, Caspase3- and MPO-positive cells and in the CLP group were also significantly increased (compared with that of the sham group) (Fig. 2e, f). While sh-SNHG16 attenuated the above pathological changes in rat lungs (*P* < 0.05, Fig. 2e, f). Afterward, the W/D value was measured to analyze the pulmonary edema. Interestingly, the W/D value of the CLP and the CLP + sh-NC group was higher than that of the Sham group, while the W/D value of the CLP + sh-SNHG16 group was lower than that in the CLP + sh-NC group (*P* < 0.05, Fig. 2g). Next, the arterial blood gas was analyzed, and it was found that down-regulating SNHG16 inhibited the CLP-induced decrease of PH and PaO_2_ and the up-regulation of PaCO_2_ (*P* < 0.05, Fig. 2h, j). Collectively, the above findings confirmed that knocking down SNHG16 alleviated CLP-induced lung cell injury, apoptosis, edema, and respiratory dysfunction in rats.

### The SNHG16-knockdown weakened the sepsis-mediated inflammation

**Fig. 3:**
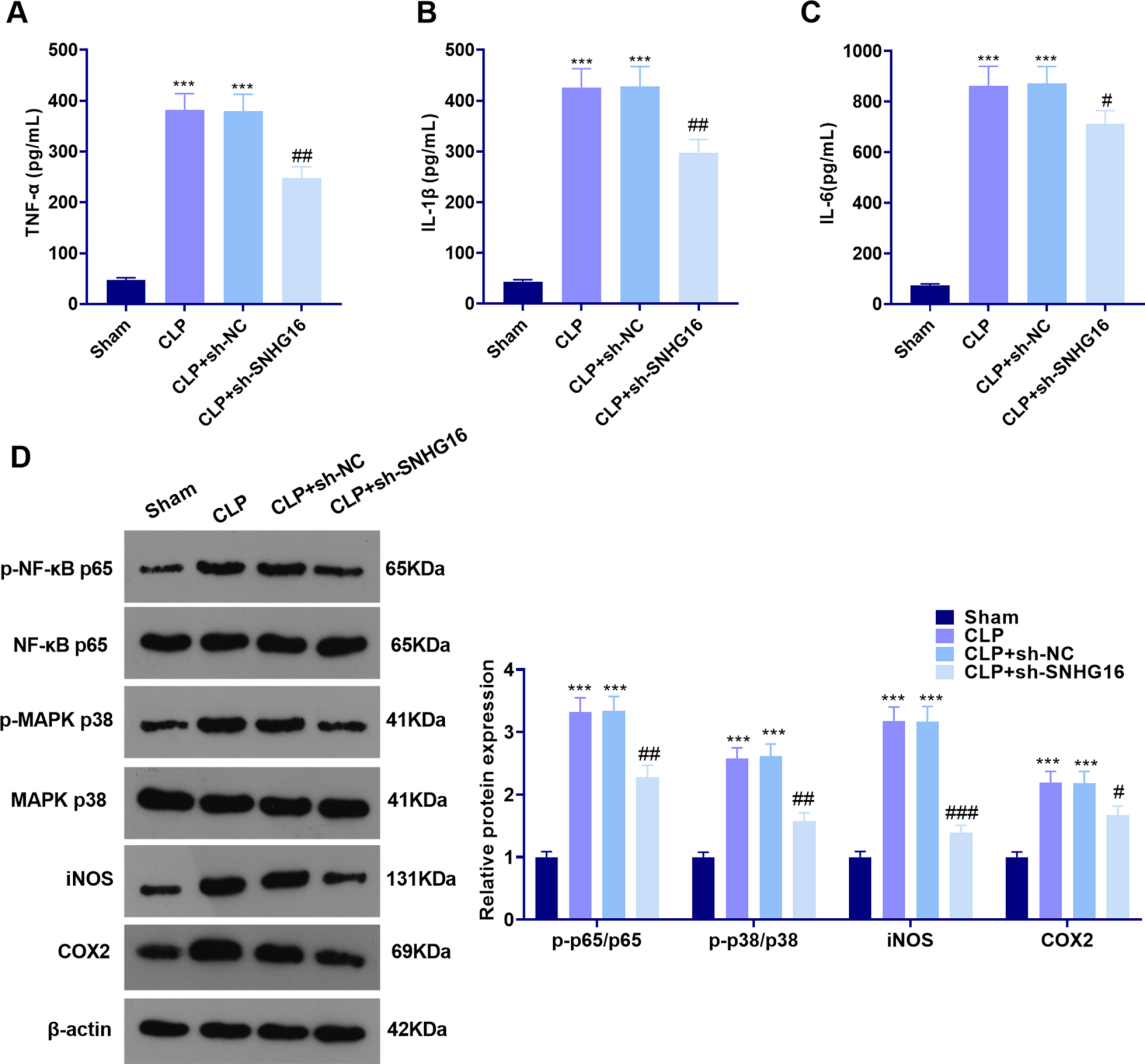
SNHG16 knockdown repressed sepsis-mediated inflammation. sh-SNHG16 was used for establishing a SNHG16-knockdodwn rats, which were then subjected to CLP to induce a sepsis model. **a**–**c** ELISA was employed to test the levels of the pro-inflammatory factors (including TNF-α, IL-1β, and IL-6); **d** WB was implemented to monitor the levels of the pro-inflammatory proteins NF-κB, MAPK-P38, iNOS and COX2 in the lung tissues. ***P < 0.001 (vs. Sham group); #*P* < 0.05; ^##^*P* < 0.01; ^###^*P* < 0.001 (vs. CLP + sh-NC group). N = 5. All experiments were repeated for three times

Furthermore, we studied the related inflammatory response after CLP induced sepsis in rats. The expression of the pro-inflammatory factors in the lung tissue of septic rats was detected by ELISA. The results suggested that TNF-α, IL-1β and IL-6 were up-regulated in the CLP group and the CLP + sh-NC group (compared with that in the Sham group). In contrast, they were down-regulated in the CLP + sh-SNHG16 group with lower level of SNHG16 (compared with that in the CLP + sh-NC group, *P* < 0.05, Fig. 3a–c). Next, we tested the levels of pro-inflammatory proteins through WB. The results illustrated that the levels of p-NF-κB p65/NF-κB p65, p-MAPK p38/MAPK p38, iNOS and COX2 in the CLP group and CLP + sh-NC group were notably higher than those in the Sham group, and their levels in the CLP + sh-SNHG16 group were lower than those in the CLP + sh-NC group (*P* < 0.05, Fig. 3d), suggesting that the SNHG16 knockdown attenuated the CLP-induced pro-inflammatory responses.

### Inhibiting SNHG16 eased lung epithelial cell damage

**Fig. 4:**
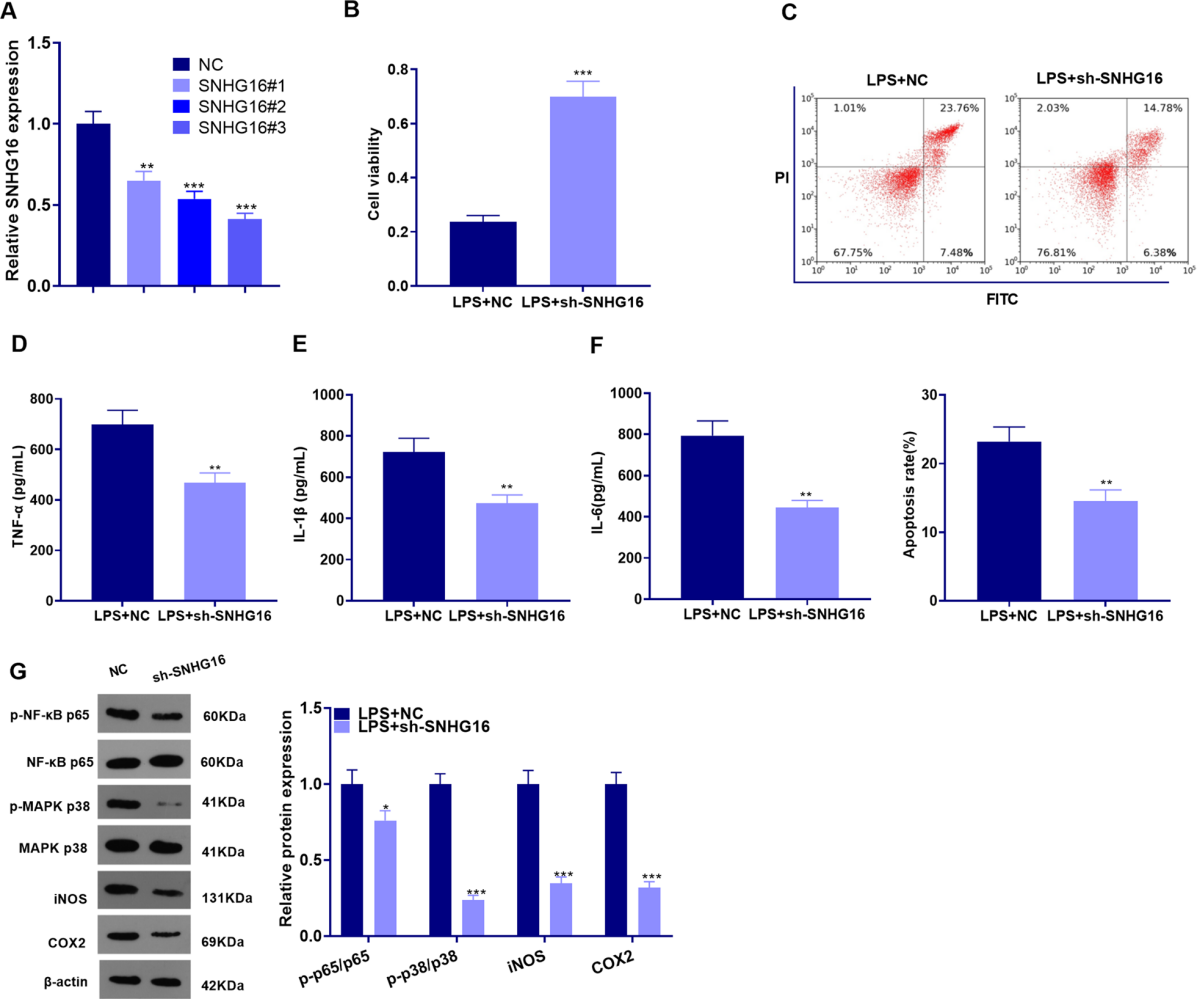
Inhibiting SNHG16 protected against LPS-induced lung cell damage. Normal human lung epithelial cell line BEAS-2B were transfected with sh-SNHG16 and then treated with LPS (100 ng/ml) for 24 h. **a** qRT-PCR was employed to examine the expression of SNHG16 in BEAS-2B cells transfected with sh-NC or sh-SNHG16; **b**, **c** MTT assay and flow cytometry were adopted to examine cell proliferation and apoptosis, respectively; **d**–**f** The expression of pro-inflammatory factors TNF-α, IL-1β and IL-6 was compared by ELISA in the culture medium of BEAS-2B cells; **g** WB was carried out to verify the levels of the pro-inflammatory proteins p-NF-κB, p-MAPK-P38, iNOS and COX2 in BEAS-2B cells. **P < 0.01; **P < 0.01; ***P < 0.001 (vs. NC group). N = 3. All experiments were repeated for three times

We constructed an SNHG16 knockdown model in the BEAS-2B cell line (*P* < 0.05, Fig. 4a) and test cell viability and apoptosis with MTT assay and flow cytometry, respectively. As a result, knocking down SNHG16 not only promoted cell proliferation but also repressed apoptosis (*P* < 0.05, Fig. 4b, c). ELISA demonstrated that the expression of TNF-α, IL-1β and IL-6 in the BEAS-2B cells was distinctly lower than those in the LPS + sh-SNHG16 group (compared with that in the LPS + sh-NC group, *P* < 0.05, Fig. 4d–f). WB showed that compared with the LPS + sh-NC group, knocking down SNHG16 repressed p-NF-κB p65/NF-κB p65, p-MAPK P38/MAPK P38, iNOS and COX2 expressions (*P* < 0.05, Fig. 4g). Thus, knocking down SNHG16 dampened the apoptosis and inflammation of BEAS-2B cells, while enhanced cell viability.

### SNHG16 up-regulated HMGB3 by competitively sponging miR-128-3p

**Fig. 5:**
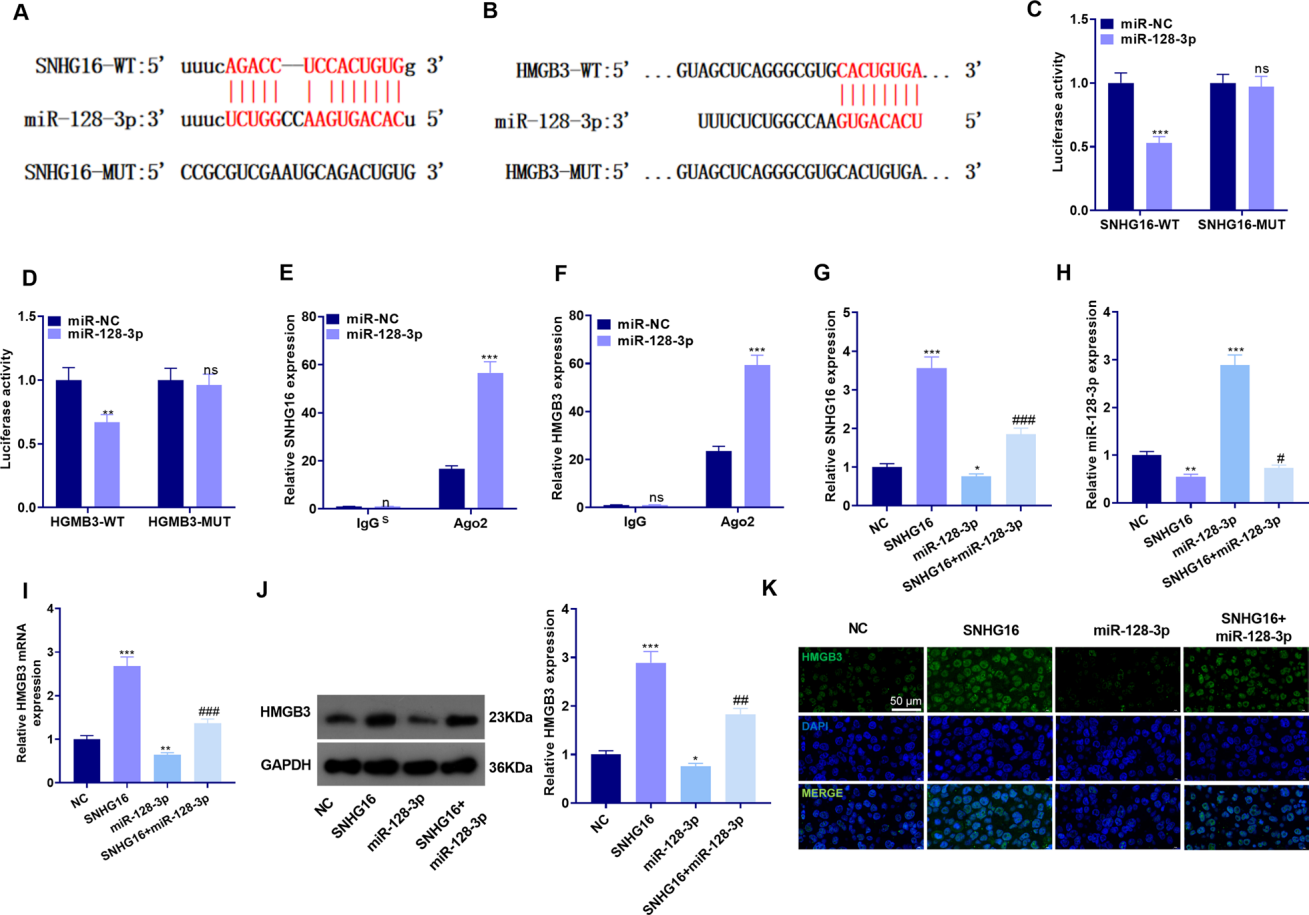
SNHG16 up-regulated HMGB3 through competitive inhibition of miR-128-3p. **a**, **b** The base binding relationships between SNHG16 and miR-128-3p, miR-128-3p and HMGB3 were queried through the Starbase and TargetScan database. **c**, **d** The dual-luciferase reporter assay was conducted to testify the binding relationship between SNHG16 and miR-128-3p, miR-128-3p and HMGB3, NS *P* > 0.05; **P < 0.01; ***P < 0.001 (vs. miR-NC group); **e**, **f** RIP assay was implemented to verify the binding relationship between SNHG16, miR-128-3p and HMGB3, NS *P* > 0.05; **P < 0.01; ***P < 0.001 (vs. miR-NC group); BEAS-2B cells were transfected with SNHG16 overexpression plasmids and/or miR-128-3p mimics. **g**–**i** The expression of SNHG16, miR-128-3p and HMGB3 in BEAS-2B cells was monitored by qRT-PCR; **j**, **k** WB and cellular Immunofluorescence were conducted to detect the level of HMGB3 protein in BEAS-2B cells. *P < 0.05; **P < 0.01; ***P < 0.001 (vs. NC group); ^#^*P* < 0.05; ^##^*P* < 0.01; ^###^*P* < 0.001 (vs. SNHG16 group). N = 3. All experiments were repeated for three times

We studied the downstream target of SNHG16 to investigate the specific mechanism of SNHG16 in sepsis-induced ALI. By querying the Starbase (http://starbase.sysu.edu.cn/) and TargetScan (http://www.targetscan.org/vert_72/) database, we discovered that miR-128-3p was an essential downstream target of SNHG16, and there were base-pairing sites between HMGB3 and miR-128-3p (Fig. 5a, b). In addition, the dual-luciferase reporter assay illustrated that the miR-128-3p mimic transfection abated the luciferase activity of SNHG16-WT and HMGB3-WT, but had no obvious inhibitory effect on that of SNHG16-MUT and HMGB3-MUT (*P* < 0.05, Fig. 5c, d). The RIP experiment showed that after transfection of miR-128-3p mimics, the amount of Ago2 precipitation in the cells was higher than that in the IgG group (*P* < 0.05, Fig. 5e, f). Moreover, qRT-PCR demonstrated that compared with the NC group, overexpressing SNHG16 up-regulated SNHG16 and HMGB3, and down-regulated miR-128-3p. In contrast, the miR-128-3p mimic transfection inhibited the up-regulation of SNHG16 and HMGB3 induced by SNHG16 overexpression (*P* < 0.05, Fig. 5g–i). Furthermore, WB and cellular Immunofluorescence also revealed that overexpressing SNHG16 up-regulated HMGB3, while the transfection of miR-128-3p mimics dampened the up-regulation of HMGB3 induced by SNHG16 (*P* < 0.05, Fig. 5j, k). The above findings confirmed that SNHG16 up-regulated HMGB3 by targeting miR-128-3p.

### SNHG16 aggravated LPS-mediated lung epithelial cell injury by modulating the miR-128-3p/HMGB3 axis

**Fig. 6:**
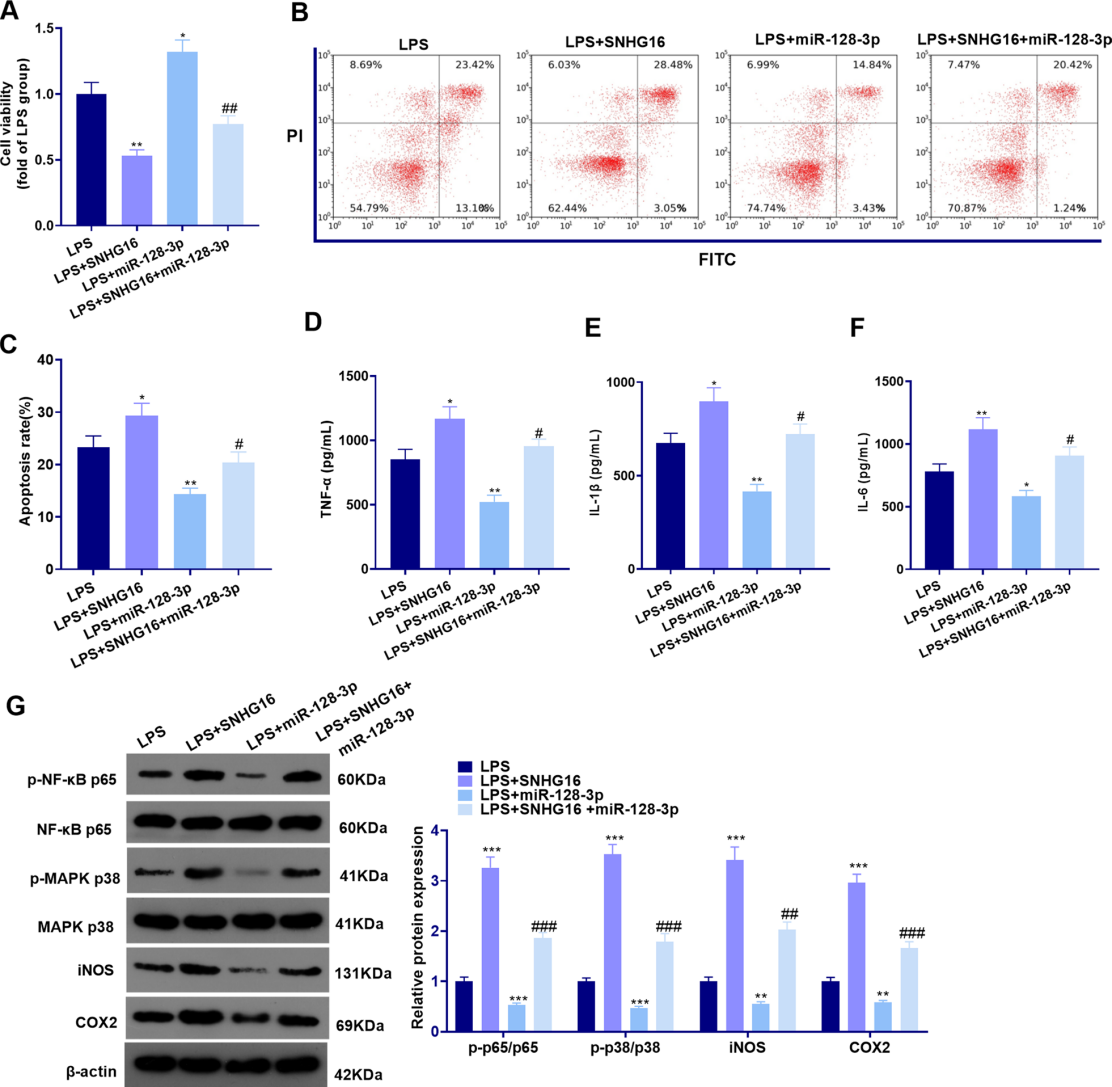
SNHG16 affected the LPS-mediated lung epithelial cell injury by modulating the miR-128-3p/HMGB3 axis. Normal human lung epithelial cell line BEAS-2B were transfected with SNHG16 overexpression plasmids and/or miR-128-3p mimics and then treated with LPS (100 ng/ml) for 24 h. **a** MTT assay was performed to examine cell viability; **b**, **c** Flow cytometry was employed to monitor apoptosis; **d**–**f** The expression of TNF-α, IL-1β, and IL-6 in cells was compared by ELISA; **g** WB was conducted to compare the levels of the pro-inflammatory proteins NF-κB, MAPK-P38, iNOS and COX2. *P < 0.05; **P < 0.01; ***P < 0.001 (vs. LPS group); ^#^*P* < 0.05; ^##^*P* < 0.01; ^###^*P* < 0.001 (vs. LPS + SNHG16 group). N = 3. All experiments were repeated for three times

Next, we studied the function of the SNHG16/miR-128-3p/HMGB3 axis in vitro. LPS was adopted to treat BEAS-2B cells, and SNHG16 overexpression plasmids and/or miR-128-3p mimics were transfected on this basis MTT assay and flow cytometry were implemented to analyze cell viability and apoptosis. The results confirmed that upregulating SNHG16 attenuated cell viability and promoted apoptosis, while miR-128-3p mimics had the opposite effects (Fig. 6a–c). Additionally, the miR-128-3p mimic transfection inhibited the SNHG16-induced decrease in cell viability and increase in apoptosis (*P* < 0.05, Fig. 6a–c). Besides, the expression of the pro-inflammatory factors was verified by the ELISA kit. The results proved that the levels of TNF-α, IL-1β and IL-6 were elevated in the LPS + SNHG16 group (compared with LPS group), while they were repressed in the LPS + miR-128-3p group compared with that in the LPS group. In contrast, the levels of TNF-α, IL-1β and IL-6 in the LPS + SNHG16 + miR-128-3p group were attenuated in comparison with that in the LPS + SNHG16 group (*P* < 0.05, Fig. 6d–f). Finally, WB showed that the levels of p-NF-κB p65/NF-κB p65, p-MAPK P38/MAPK, iNOS and COX2 in the LPS + SNHG16 group were higher than those in the LPS group, while their levels in the LPS + miR-128-3p were lower than those in the LPS group. Meanwhile, the levels of p-NF-κB p65/NF-κB p65, p-MAPK P38/MAPK, iNOS, and COX2 were lower in the LPS + SNHG16 + miR-128-3p group than that in the LPS + SNHG16 group (*P* < 0.05, Fig. 6g). Thus, SNHG16 aggravated lung cell damage and inflammation by regulating the miR-128-3p/HMGB3 axis.

## Conclusions

Sepsis can cause life-threatening multiple organ dysfunction, of which ALI and acute respiratory distress are the earliest and most common symptoms. The main pathological manifestations of ALI are inflammatory cell infiltration, lung epithelial cell damage, endothelial cell destruction, pulmonary interstitial congestion and edema [23]. Due to the complex pathological mechanism and the rapid progress of ALI, there is still a lack of effective clinical countermeasures. Our study proved that SNHG16 is up-regulated in septic ALI rats, and down-regulating SNHG16 eases lung injury by modulating the miR-128-3p/HMGB3 axis.

LncRNAs are abundant in mammals. They modulate gene expression at the transcriptional and post-transcriptional levels and contribute to diversified biological processes such as chromatin remodeling, histone modification, and cell cycle regulation [24]. Over the years, several studies have shown that lncRNAs intervene inflammation by modulating inflammatory mediators. Some scholars discovered that MALAT1 is up-regulated in rat tissues with septic lung injury, and knocking down MALAT1 ameliorates the lung injury in rats by attenuating the MAPK p38/NF-κB p65 signaling pathway [25]. SNHG16 is a novel lncRNA, which was first studied by Christensen et al. Their findings showed that SNHG16 is up-regulated in colorectal cancer cell lines, and knocking down SNHG16 inhibits cell proliferation and migration, and induces apoptosis [26]. Also, the carcinogenic effect of SNHG16 is proved in NSCLC [22]. Besides, SNHG16 is abnormally expressed in other lung diseases, including ALI. For instance, Zhang et al. hold that SNHG16 up-regulates IGF2 by targeting miR-370-3p, thereby preventing LPS from inhibiting lung cell viability and promoting cell apoptosis and inflammation [10]. Moreover, Zhou et al. confirmed that SNHG16 is up-regulated in the serum of ALI patients, and knocking down SNHG16 in the LPS-treated lung cell injury model inhibits the expression of CC motif chemokine ligand 5 (CCL5) by sponging miR-146a-5p, thereby reducing the inflammatory injury of lung cells [9]. Thus, knocking down SNHG16 may be a new treatment for ALI. Fortunately, our research results suggested that knocking down SNHG16 abates sepsis-mediated lung injury and dysfunction, reduces pulmonary edema and inflammation, improves lung cell viability, and prevents cell apoptosis.

Accumulating researches have proved that miRNAs contribute to regulating sepsis-induced ALI. For example, Cao et al. reported that miR-145 targets TGFBR2 and alleviates sepsis-induced ALI [27]. In addition, Pan et al. also showed that miR-124 reduces the ALI symptoms in septic mice by down-regulating MAPK14 and inactivating MAPK signaling pathway [28]. The literature also revealed that miR-98 negatively regulates HMGA2 by inhibiting the NF-κB signaling pathway to protect mice from sepsis-mediated lung damage [29]. Still, we are very curious whether miR-128-3p can treat sepsis-induced ALI by regulating the downstream genes. Ding et al. took the lead in research and proved that miR-128-3p promotes the SOD expression while down-regulates MPO and MDA in septic mouse tissues. Meanwhile, miR-128-3p enhances the protection of dexmedetomidine against ALI in sepsis mice by targeting and dampening MAPK14 [15]. Above all, some studies have found that down-regulated SNHG16 inhibits HOXA7 by sponging miR-128-3p to prevent the development of glioblastoma [30]. Here, we discovered that miR-128-3p is a vital downstream target of SNHG16 through bioinformatics, and we confirmed that miR-128-3p is negatively regulated by SNHG16. Meanwhile, the miR-128-3p mimic transfection represses the LPS-induced apoptosis and enhancement of inflammation and reduces lung injury.

Studies have shown that miRNAs bind to the 3′-UTR of their target mRNAs and reduce their expression and contribute to gene regulation. HMGB3, a multifunctional protein and a crucial downstream target of various miRNAs, is involved in the occurrence and progression of diversified malignancies. Yamada et al. found that the targeted inhibition of HMGB3 by miR-205-5p reduces the aggressiveness of prostate cancer [31]. Some scholars have found that overexpressing miR-758 targeting HMGB3 inhibits the malignant biological behaviors and strengthens NSCLC cell apoptosis [32]. However, whether HMGB3 plays a role in sepsis-induced organ inflammation remains elusive. Biological information analysis demonstrated that miR-128-3p targets and inhibits HMGB3, and overexpressing HMGB3 counteracts miR-128-3p. Namely, overexpressing HMGB3 partially eliminates the protective effect of miR-128-3p on LPS-damaged lung cells, promotes cell apoptosis, inhibits cell viability, and activates inflammation.

Overall, SNHG16 is up-regulated in sepsis-mediated ALI. In addition, inhibiting SNHG16 targets miR-128-3p and up-regulates HMGB3, thereby reducing lung injury, pulmonary edema and inflammation, and increasing lung cell viability and preventing cell apoptosis. Nevertheless, we only conducted male animal experiments in this study. In the future, more researches need to be conducted: (1) To confirm the regulatory axis of SNHG16/miR-128-3p/HMGB3 in larger number of female and male rats; (2) To evaluated the expressions of SNHG16/miR-128-3p/HMGB3 in ALI patients with sepsis.

## Supplementary Information

**Additional file 1**. The original gels in figure 2C and 3D.

**Additional file 2**. The original gels in figure 4G.

**Additional file 3**. The original gels in figure 5J and 6G.

## Data Availability

The data sets used and analyzed during the current study are available from the corresponding author on reasonable request.
