# Supplementary figures and images for: Down-regulation of SNHG16 alleviates the acute lung injury in sepsis rats through miR-128-3p/HMGB3 axis
Source: BMC Pulm Med. 2021 Jun 6;21:191. doi: 10.1186/s12890-021-01552-0 (PMC8180123; doi:10.1186/s12890-021-01552-0)

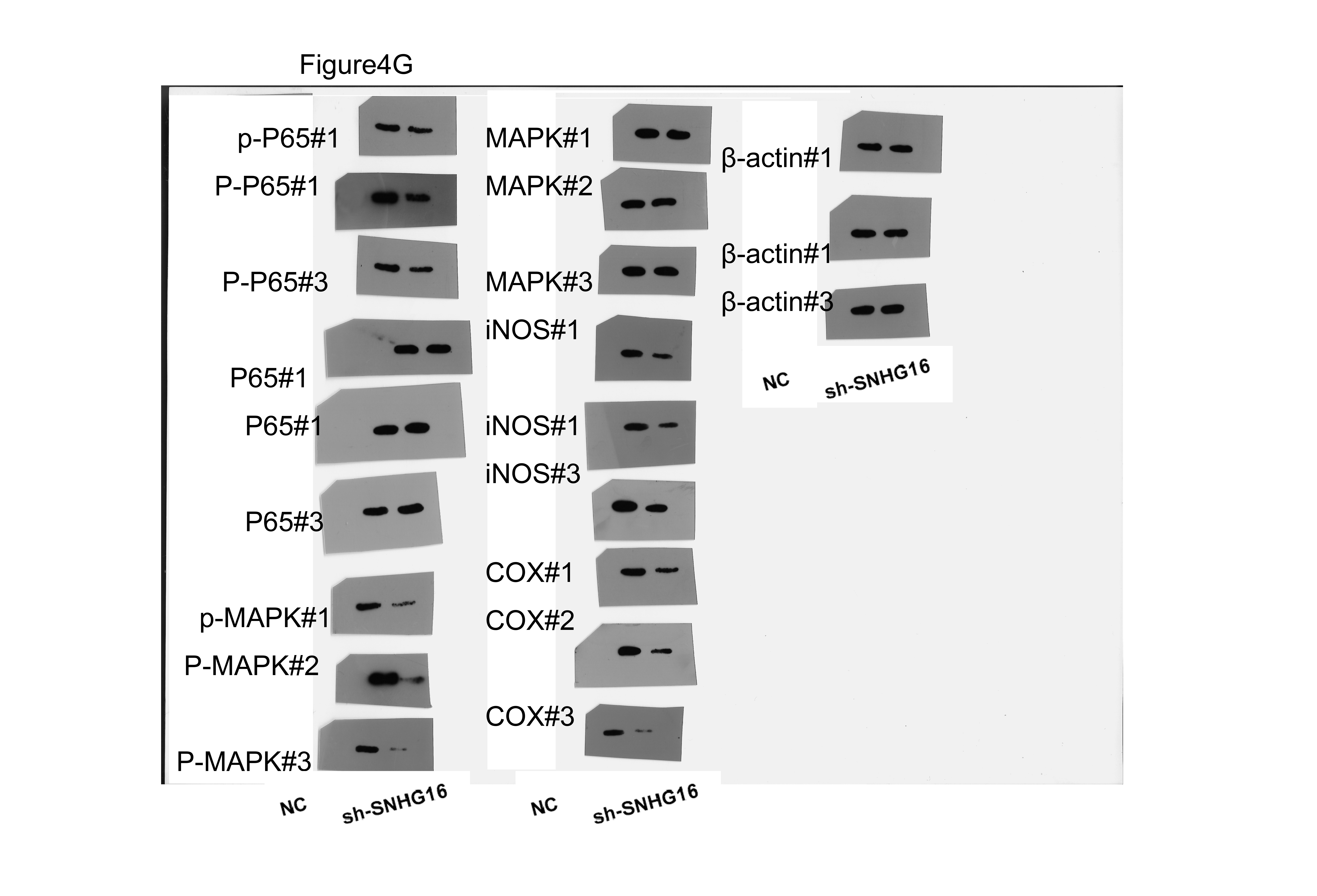

Supplement: Supplementary file 2 — Additional file 2. The original gels in figure 4G. [file 12890_2021_1552_MOESM2_ESM.jpg]
